# Hydrogen Sulfide Reduces Cognitive Impairment in Rats After Subarachnoid Hemorrhage by Ameliorating Neuroinflammation Mediated by the TLR4/NF-κB Pathway in Microglia

**DOI:** 10.3389/fncel.2020.00210

**Published:** 2020-07-09

**Authors:** Hongzhou Duan, Liang Li, Shengli Shen, Yuanyuan Ma, Xiangdong Yin, Zhen Liu, Changwei Yuan, Yingjin Wang, Jiayong Zhang

**Affiliations:** ^1^Department of Neurosurgery, Peking University First Hospital, Beijing, China; ^2^Laboratory Animal Center, Peking University First Hospital, Beijing, China

**Keywords:** subarachnoid hemorrhage, cognitive impairment, hydrogen sulfide, neuroinflammation, TLR4/NF-κB pathway

## Abstract

**Background and Aims**: Cognitive impairment is one of the major complications of subarachnoid hemorrhage (SAH) and is closely associated with neuroinflammation. Hydrogen sulfide (H_2_S) has been shown to have an anti-inflammatory effect and reduce cognitive impairment in neurodegenerative diseases, but its effects in SAH have been little studied. This study aimed to investigate the effects of H_2_S on cognitive impairment after SAH and the possible underlying mechanisms.

**Methods**: Forty-eight male Sprague–Dawley (SD) rats were randomly divided into three groups: a sham group, a SAH group, and a SAH + NaHS (an H_2_S donor) group. The endovascular perforation technique was used to establish the experimental SAH model. NaHS was administered intraperitoneally. An active avoidance test (AAT) was performed to investigate cognitive function. The expression of TNF-α, toll-like receptor 4 (TLR4), and NF-κB p65 in the hippocampus was measured by Western blot and immunohistochemistry. The types of cells expressing TNF-α were detected by double immunofluorescence staining.

**Results**: Compared to that in the sham group, the learning and memory ability of rats in the SAH group was damaged. Furthermore, the expression of TNF-α, TLR4, and NF-κB p65 in the hippocampus was elevated in the SAH group (*p* < 0.05). TNF-α was mainly expressed in activated microglia, which was consistent with the expression of TLR4. Treatment with NaHS significantly decreased the cognitive impairment of rats after SAH and simultaneously reduced the expression of TNF-α, TLR4, and NF-κB p65 and alleviated the nuclear translocation of NF-κB p65 (*p* < 0.05).

**Conclusions**: The neuroinflammation reaction in microglia contributes to cognitive impairment after SAH. H_2_S reduced the cognitive impairment of rats after SAH by ameliorating neuroinflammation in microglia, potentially *via* the TLR4/NF-κB pathway.

## Introduction

Subarachnoid hemorrhage (SAH) accounts for 5–9% of all strokes but is responsible for 30% of stroke-related mortality (El Amki et al., 2018) and affects people younger than those who suffer ischemic strokes. With the improvements in diagnosis and treatment, the mortality of SAH patients is decreasing, and the functional prognosis of survivors is attracting increasing attention (Macdonald and Schweizer, 2017). Cognitive impairment, one of the major complications of SAH, mainly manifests as dysfunctions of memory, executive function, and language (Etminan and Macdonald, 2017). A study showed that 73% of SAH patients experienced cognitive impairment (Wong et al., 2012), greatly impacting patient quality of life and social functioning and imposing a tremendous social burden. Although various risk factors may be associated with cognitive impairment after SAH, including cerebral edema, infarction, hydrocephalus, delayed cerebral ischemia and reduced hippocampal volume, the underlying mechanisms remain unclear, and no effective therapies have yet been developed.

Inflammation has been proven to be a driving force in cognitive decline in neurodegenerative diseases such as Alzheimer’s disease (AD), and its progress could be arrested by anti-inflammatory agents (McGeer et al., 2016). Several studies have demonstrated that inflammation is also involved in the pathogenesis of cognitive impairment after SAH and that neutrophil depletion after SAH could ameliorate this deficit (Provencio et al., 2016), but related studies are scarce. Neuroinflammation after SAH is generally triggered by the breakdown and degradation of red blood cells, which leads to the activation of microglia, the macrophage-like cells of the central nervous system (CNS). An important contributor to microglial activation is toll-like receptor 4 (TLR4) which is mainly expressed on glia, primarily microglia (Buchanan et al., 2010; Lucke-Wold et al., 2016). TLRs play crucial roles in the innate immune system by recognizing pathogen-associated molecular patterns (PAMPs) and damage-associated molecular patterns (DAMPs) and lead to activation of the NF-κB transcription factors. These transcription factors regulate the expression of cytokines and chemokines such as TNF-α (Kawasaki and Kawai, 2014). The TLR4/NF-κB signaling pathway may be implicated in neuroinflammation after SAH and blockage of TLR4 can alleviate cognitive impairment and secondary brain damage after SAH (Wang et al., 2013; Provencio et al., 2016).

As an accepted third gasotransmitter, (H_2_S) is the focus of a growing body of research on its neuroprotective effects. Several studies indicate that H_2_S could ameliorate neuroinflammation and learning and memory impairment (Xuan et al., 2012), but the effects of H_2_S in SAH, particularly on cognitive impairment after SAH, remain unknown. This study investigated the hypothesis that H_2_S can reduce cognitive impairment after SAH, potentially by ameliorating neuroinflammation mediated by the TLR4/NF-κB pathway in the brain.

## Materials and Methods

### Animals

All experiments were approved by the Ethics Committee of Peking University First Hospital and conducted following the guidelines of the National Institutes of Health on the care and use of animals. Healthy adult male Sprague–Dawley (SD) rats (280–300 g) were purchased from SPF (Beijing) Biotechnology Company Limited and housed under standard laboratory conditions (temperature- and humidity-controlled animal quarters with a 12-h light/dark cycle) with free access to food and water.

### SAH Model

An endovascular perforation model was established as previously reported (Bederson et al., 1995). Briefly, rats were anesthetized with ketamine (166 mg/kg IP) after the intramuscular administration of atropine (0.02 mg/kg) to antagonize ketamine’s side effects. The right carotid artery, external carotid artery (ECA) and internal carotid artery (ICA) were identified and dissected. A 4–0 monofilament suture sharpened at one end was introduced through the ECA, past the common carotid bifurcation, and into the ICA. The suture was advanced through the ICA until its intracranial bifurcation where resistance was felt and then was pushed 3 mm further, penetrating the artery. Then, the suture was withdrawn, and the SAH model was produced. In the sham group, the suture was inserted but withdrawn before the resistance was felt such that the intracranial ICA was not perforated.

### Drug Administration and Experimental Design

Forty-eight SD rats were randomly divided into three groups of 16 rats each: a sham group, a SAH group, and a SAH + NaHS (sodium hydrosulfide, and H_2_S donor) group. Rats that died during the operation or drug administration were replaced by new rats to ensure 16 rats per group in the experiment. In the SAH + NaHS and SAH groups, NaHS (5.6 mg/kg) and an equal volume of saline, respectively, were injected intraperitoneally once daily beginning at 2 days before surgery for 4 days.

### Neurological Scoring

Neurological evaluations of the rats in each group were carried out at 24 h and on day 4 after surgery by a colleague who was “blinded” to the rats’ treatments. To assess neurological deficits, an established neurological scoring system (Garcia et al., 1995) was used, which consisted of six test items: spontaneous activity (in the cage for 5 min; 0–3), the symmetry of movements (four limbs; 0–3), the symmetry of forelimbs (outstretching while held by the tail; 0–3), climbing the wall of the wire cage (1–3), reaction to touch on either side of the trunk (1–3), and response to vibrissae touch (1–3). Total scores ranged from 3 to 18; a lower score indicates worse neurological function. Data on the first four items, associated with the motor function (motor function score), were extracted to evaluate the motor performance of rats and compare it among the groups.

### Active Avoidance Test

Learning and memory ability was evaluated by an active avoidance test (AAT; Cheng et al., 2011). The rats from each group were tested in an automated two-way shuttle-box apparatus (purchased from Jiangsu SANS Biological Technology Company Limited), which was divided into two equally sized compartments (30*30*25 cm) connected by a gate. Compartment floors were independently electrifiable, and the unconditioned stimulus (US) was a 2 mA scrambled electrical footshock delivered through the grid floor. The conditioned stimulus (CS) was a light that was switched on alternately in the two compartments. The CS was presented for 5 s and was immediately followed by the US. The US terminated automatically if the rat crossed over to the other compartment and lasted for 10 s at maximum. Briefly, a learning session of four consecutive days was conducted from the 5th day after surgery (and designated Day 1 to Day 4). On each day, 40 consecutive trials with 20 s intertrial intervals (ITI) were conducted with each rat. Each rat was allotted a 5-min adaptation period before the first trial of each day. The following parameters were recorded automatically: (1) number of active avoidance responses (AARs; crossing to the other compartment during the CS); (2) number of errors (Es; failing in crossing to the other compartment during both CS and US); and (3) number of ITI responses (ITIRs; crossing during the ITI stage). Learning and memory ability was assessed as the ratio of AAR number to the total number of trials (expressed as a percentage, AAR%) and the ratio of E number to the total number of trials (E%) Locomotor activity was assessed as the ratio of the ITIR number to the total trial number (ITIR%), which reflected rat motor function.

### SAH Grade

The SAH grades of all rats in each group were quantified to evaluate the bleeding scale when they were sacrificed to estimate SAH severity following the published grading system based on the volume of blood clots in six parts of the basal cisterns (Sugawara et al., 2008). The final scores, ranging from 0 to 18, were obtained by adding the six scores from the different parts, each of which ranged from 0 to 3. A higher score indicates more severe SAH.

### Western Blot Analysis

Expression of TNF-α, NF-κB p65, and TLR-4 was analyzed by Western blot. Eight rats per group were sacrificed by rapid decapitation at 48 h after surgery. The brains were removed, and the hippocampi were separated on ice. Part of each hippocampus was rapidly frozen in liquid nitrogen. The hippocampal samples were homogenized, and proteins were extracted by RIPA buffer. Protein concentrations were determined using a BCA protein assay kit (Thermo Fisher Scientific Inc., Waltham, MA, USA). The proteins were then separated by SDS-polyacrylamide gel electrophoresis (SDS-PAGE) using 12% acrylamide gels and transferred onto a polyvinylidene difluoride (PVDF) membrane. The membrane was then incubated with the following primary antibodies at 4°C overnight: anti-TLR4 antibody (1:500, ab 13556, Abcam), anti-NF-κB p65 antibody (1:2,000, ab16502, Abcam), anti-TNF-α antibody (1:500, ab6671, Abcam). Anti-ACTB antibody (1:2,000, Sangon Biotech) was used as an internal control. After incubation with horseradish peroxidase (HRP)-conjugated goat anti-rabbit secondary antibody (1:2,000, ZB02301, ZSGB-BIO) for 2 h at room temperature, the blotted protein bands were visualized by enhanced chemiluminescence (ECL) Western blotting detection kit (P0018FS, Beyotime).

### Histopathologic Examination

After decapitation, parts of the brains and hippocampus were removed and placed in paraformaldehyde at 4°C overnight for subsequent hematoxylin and eosin (H&E) staining, immunohistochemistry, and immunofluorescence imaging. For histological examination, the fixed brain samples were dehydrated with gradient ethanol and processed for routine paraffin embedding. Coronal cuts of brains were then sectioned into 4 μm thick sections at approximately 6 mm anterior to the groove between forebrain and cerebellum and stained with H&E for visualization of the hippocampus (Jeon et al., 2010). The morphology of hippocampus areas, including the dentate gyrus (DG) and CA1–3, was observed by a light microscope.

### Immunohistochemistry

Immunohistochemistry of paraffin-embedded sections was performed to determine the immunoreactivity of TLR4, NF-κB p65, and TNF-α. The sections were deparaffinized, rehydrated, quenched with 3% H_2_O_2_ for blocking endogenous peroxidase activity, and washed in PBS before antigen retrieval by microwaving in citrate buffer. Following the application of blocking serum, the sections were incubated in the following primary antibodies overnight at 4°C: anti-TLR4 antibody (1:50, ab 13556, Abcam), anti-NF-κB p65 antibody (1:300, ab16502, Abcam), anti-TNF-α antibody (1:100, ab6671, Abcam). After being washed for 15 min in PBS, the sections were incubated with HRP-conjugated goat anti-rabbit secondary antibody (1:200, ZB02301, ZSGB-BIO) for 50 min at room temperature. DAB was used, and counterstaining was performed with hematoxylin. The slides were then dehydrated and mounted in mounting medium. Microscopy of the sections was performed by a “blinded” pathologist, and the mean density (IOD/area) of identical regions of interest in the hippocampus across eight microscope fields (400× magnification) was determined by Image-Pro Plus 6.0. Nuclear translocation of NF-κB p65 was assessed, and the IOD/nuclear area was compared among the groups.

### Immunofluorescence Staining

Immunofluorescence colocalization analysis was performed to examine the expression of TLR4 and TNF-α in brain cells and the expression of TNF-α in microglia. The paraffin-embedded 4-μm sections were deparaffinized and rehydrated before antigen retrieval by microwaving. Following the application of blocking serum, the sections were incubated with the following primary antibodies overnight at 4°C: anti-TLR4 antibody (1:100, ab22048, Abcam), anti-TNF-α antibody (1:100, ab6671, Abcam), anti-IBA-1 antibody (1:500, GB12105, Servicebio) and anti-TNF-α antibody (1:100, ab6671, Abcam). The sections were then incubated with Alexa Fluor 488-conjugated goat anti-mouse secondary antibody (1:400, GB25301, Servicebio) and CY3-conjugated goat anti-rabbit secondary antibody (1:300, GB21303, Servicebio) for 50 min at room temperature, rinsed, counterstained with DAPI and air-dried before mounting. Fluorescence was imaged by fluorescence microscope. The number of TNF-α/TLR-4 and TNF-α/IBA-1 co-positively immunostained cells in each section of identical regions of interest was determined in eight high-power fields (HPFs; 400× magnification) and defined as cell density (cells/HPF).

### Statistical Analysis

All data were presented as the mean ± SD, and SPSS 20.0 was used for statistical analysis. For the data from the behavioral experiments comprising values measured over time (AAT), mixed-design ANOVA was performed followed by contrast analyses for evaluation of between- and within-group effects. Other variables satisfying the normal distribution assumption were analyzed by one-way ANOVA followed by the Bonferroni test for *post hoc* analysis. For nonnormally distributed variables, nonparametric comparisons between groups were performed using the Kruskal–Wallis test. *p* < 0.05 was considered statistically significant.

## Results

### Overview of Mortality and SAH Grade

**Figure 1:**
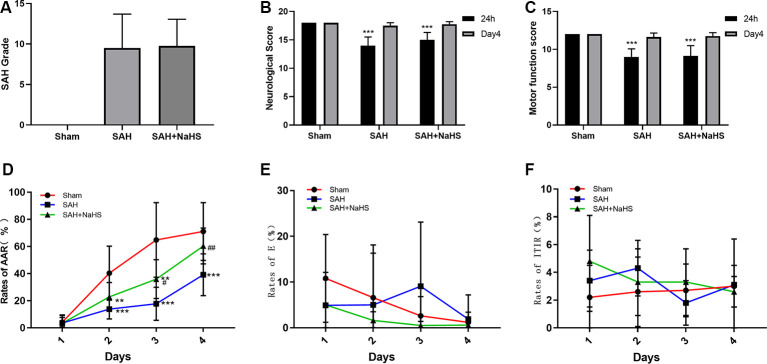
**(A)** The quantification of subarachnoid hemorrhage (SAH) grades (*n* = 8 per group, *p* = 0.978) did not significantly differ between the SAH and SAH + NaHS groups. **(B)** Neurological scores of rats at 24 h and on day 4 after surgery. **(C)** Motor function scores of rats at 24 h and on day 4 after surgery. **(D–F)** Learning performance of rats in the active avoidance test (AAT). **(D)** The SAH group showed a lower rate of active avoidance response (AAR) than the sham group, and NaHS improved the rates of AAR compared with those in the SAH group. **(E)** The error rates did not significantly differ among the three groups but tended to decrease with increasing training days in the sham and SAH + NaHS groups. **(F)** Error rates did not significantly differ among the four training days. Data are means ± SD. *n* = 8; **p < 0.01, ***p < 0.001 vs. sham group; ^#^*p* < 0.05, ^##^*p* < 0.01 vs. SAH group.

The mortality rate within 48 h after surgery was 5.9% (1/17 rats) in the sham group, 15.8% (3/19 rats) in the SAH group, and 11.1% (2/18 rats) in the SAH + NaHS group. SAH score did not significantly differ between the SAH and SAH + NaHS groups (Figure 1A).

### Neurological Deficits After SAH

The neurological scores and motor function scores of rats at 24 h after surgery were significantly lower in the SAH group than in the sham group, suggesting pronounced neurological impairment induced by SAH. This impairment presented mainly as reduced spontaneous activity and limb weakness. NaHS slightly increased the neurological and motor function scores of rats with SAH, but the differences were not significant (*p* = 0.098 and *p* = 0.792, respectively). On day 4 after surgery, the neurological function of rats in both the SAH and SAH + NaHS groups had recovered, and there was no significant difference in the neurological scores and motor function scores among the three groups, as shown in Figures 1B,C.

### Cognitive Impairment After SAH

Figure 1D depicts the mean rates of AAR of rats in the AAT. As shown in the figure, the rates of AAR in all three groups tended to increase over training days, although the mean rate of AAR within a training day differed among groups. The mixed ANOVA showed that the group factor (*F*_(2,21)_ = 12.02, *p* < 0.001) and DAY factor (*F*_(3,63)_ = 90.159, *p* < 0.001) were significant. The significant group by DAY interaction (*F*_(6,63)_ = 6.043, *p* < 0.001) indicated that the differences between groups varied depending on the training day evaluated. Pairwise comparisons demonstrated that the SAH group showed a lower rate of AAR than the sham group on days 2 (*p* < 0.001), 3 (*p* < 0.001), and 4 (*p* < 0.001). NaHS improved the rate of AAR on day 3 (*p* < 0.05) and 4 (*p* < 0.01) compared with the corresponding rates for SAH alone. Figure 1E depicts the mean error rates of rats in the AAT. The mixed ANOVA showed that the DAY factor (*F*_(3,63)_ = 3.227, *p* = 0.028) was significant, indicating that rats tended to make fewer errors with more training days. Neither the group factor (*F*_(2,21)_ = 1.095, *p* = 0.353) nor the group by DAY interaction (*F*_(6,63)_ = 1.574, *p* = 0.169) was significant. The Friedman test was then used to compare the error rates among days within each group. The results showed that error rates in the sham and SAH + NaHS groups were significantly reduced in the late training days (*p* = 0.002 and *p* = 0.011, respectively) while those in the SAH group did not significantly differ among the four training days (*p* = 0.290). Figure 1F depicts the mean rates of ITIR of rats in the AAT. The mixed ANOVA showed that the group factor (*F*_(2,21)_ = 0.642, *p* = 0.537), DAY factor (*F*_(3,63)_ = 0.940, *p* = 0.427) and group by DAY interaction (*F*_(6,63)_ = 1.475, *p* = 0.201) were all nonsignificant.

### Histopathological Characteristics of Hippocampus

**Figure 2:**
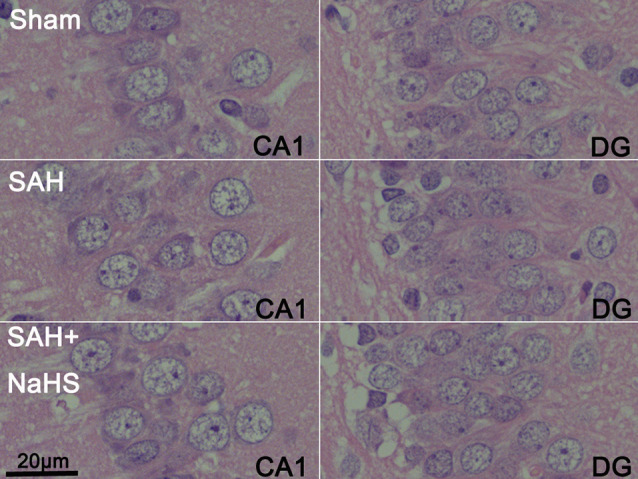
Representative micrographs of dentate gyrus (DG) and CA1 regions of brain sections stained with hematoxylin and eosin (H&E) in the sham, SAH, and SAH + NaHS groups (scale bar = 20 μm in all images).

The sections stained with H&E were examined to observe the histopathological characteristics of hippocampus regions, including DG and CA1–3, and the extents of inflammatory cell infiltration and neuronal necrosis. No neuronal necrosis or neutrophil infiltration was apparent in the three groups. Representative micrographs of the DG and CA1 regions are shown in Figure 2.

### The Expression of TLR4, NF-κB p65, and TNF-α

**Figure 3:**
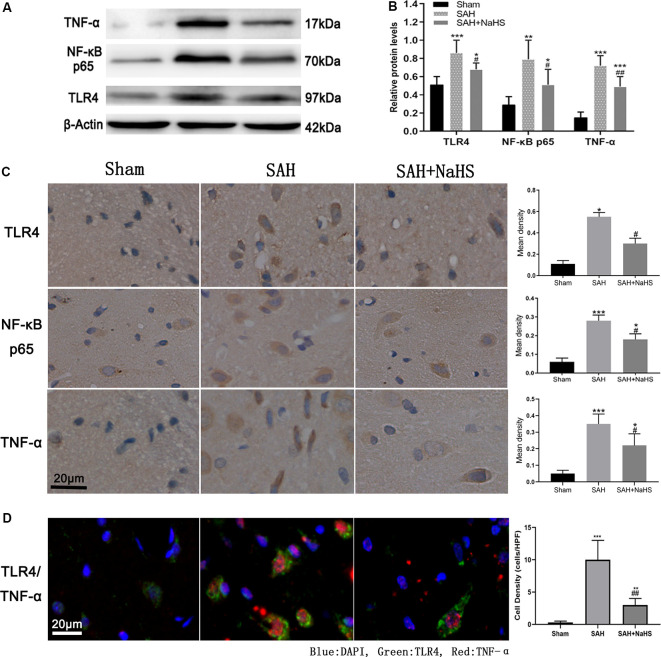
**(A,B)** Western blotting analysis of relative protein contents in the hippocampus. **(A)** Western blot of protein contents of toll-like receptor 4 (TLR4), NF-κB p65, and TNF-α in the sham, SAH, and SAH + NaHS groups. **(B)** Bar graphs showing the relative optical density normalized to β-Actin as determined by ImageJ. **(C,D)** Expression and localization of TLR4, NF-κB p65, and TNF-α. **(C)** Representative micrographs of immunohistochemistry and quantification of mean density analysis by Image-Pro Plus 6.0 showing different levels of expression of TLR4 and TNF-α in the hippocampus, and NF-κB p65 in the cell nucleus in the hippocampus in the sham, SAH, and SAH + NaHS groups (scale bar = 20 μm in all images in panel **C**). **(D)** Representative micrographs of double immunofluorescence staining; overlapped images show that the cells expressing TNF-α (red) mostly colocalized with TLR4 (green). Quantification analysis showed that the density (cells/HPF) of cells staining positively for both TLR4 and TNF-α was much lower in the SAH + NaHS group than in the SAH group (scale bar = 20 μm in all images in panel **D**). Data are means ± SD. *n* = 8; *p < 0.05, **p < 0.01, ***p < 0.001 vs. sham group; ^#^*p* < 0.05, ^##^*p* < 0.01 vs. SAH group.

The expression of TLR4, NF-κB p65, and TNF-α was initially detected by Western blot. As shown in Figures 3A,B, the hippocampus of rats in the sham group expressed the three proteins at low levels; the levels were significantly higher in the SAH group (*p* < 0.01). The expression levels of TLR4, NF-κB p65, and TNF-α in the hippocampus of the SAH + NaHS group were significantly lower than those in the hippocampus of the SAH group (*p* < 0.05).

The expression and localization of TLR4, NF-κB p65, and TNF-α were further assessed by immunohistochemistry and immunofluorescence colocalization analysis. As shown in Figure 3C, NaHS downregulated the expression of TLR4, NF-κB p65, and TNF-α (*p* < 0.05) in the hippocampus, countering the increase in expression induced by SAH. In the immunohistochemistry analysis of NF-κB p65, the IOD/area in cell nucleus was found to be significantly higher in the SAH group than in the sham group, indicating that the nuclear translocation of NF-κB was increased after SAH. However, NaHS treatment decreased the increase in IOD/area induced by SAH, indicating that NaHS inhibited the nuclear translocation of NF-κB. TLR4/TNF-α immunofluorescence colocalization analysis showed that over 90% percent of cells positively stained by TNF-α antibody were costained with TLR4 antibody, with TNF-α being expressed primarily by cells expressing TLR4. Furthermore, the density (cells/HPF) of cells staining positively for both TLR4 and TNF-α was much lower in the SAH + NaHS group than in the SAH group (Figure 3D).

### Activation of Microglia

**Figure 4:**
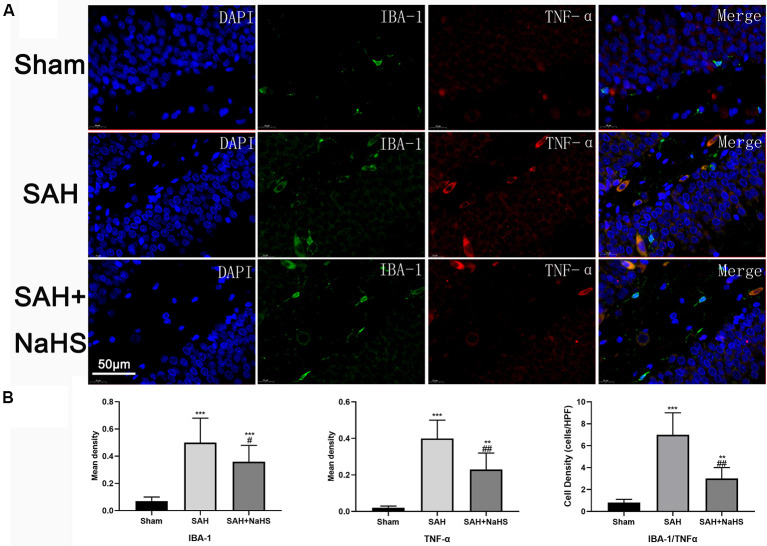
The expression and distribution of TNF-α and activation of microglia were detected by double immunofluorescence staining. **(A)** Representative micrographs of co-immunofluorescence staining. IBA-1-positive fluorescent cells (green); TNF-α-positive fluorescent cells (red); DAPI (blue). The SAH + NaHS group exhibited more ramified microglia, which stained positive for IBA-1 antibody alone, while the SAH group exhibited more amoeboid microglia with reduced ramification and enlarged cell bodies, which stained positive for both IBA-1 and TNF-α antibodies (scale bar = 50 μm in all images in panel **A**). **(B)** Quantitative analysis showed that the mean density (IOD/area) of IBA-1 and TNF-α was significantly increased in the SAH group relative to the sham group and that this increase was significantly inhibited by NaHS. The density of cells positively stained by both IBA-1 and TNF-α was significantly higher in the SAH group than in the sham group and lower in the SAH + NaHS group than the SAH group. Data are mean ± SD. *n* = 8; **p < 0.01, ***p < 0.001 vs. sham group; ^#^*p* < 0.05, ^##^*p* < 0.01 vs. SAH group.

To assess the activation of microglia in the hippocampus and gain insight into the identity of cells expressing TNF-α, hippocampus sections were co-immunostained with TNF-α antibody and an antibody against IBA-1, a specific marker for microglia/macrophages (Murakami et al., 2011). Results showed that the mean density (IOD/area) of IBA-1 was significantly increased in the SAH group and significantly reduced in the SAH + NaHS group. Furthermore, the SAH + NaHS group showed greater numbers of ramified microglia, which were positively stained for the IBA-1 antibody alone, while the SAH group showed greater numbers of amoeboid microglia with fewer ramifications, which were positively stained for both IBA-1 and TNF-α antibodies (Figure 4). Quantification analysis showed that the density of cells with positive staining for both IBA-1 and TNF-α was significantly higher in the SAH group than in the sham group and lower in the SAH + NaHS group than the SAH group.

## Discussion

Cognitive impairment is one of the major complications of SAH and manifests primarily as learning and memory deficits, which requires much more attention in the clinic. Here, we used the AAT to assess the learning and memory ability of rats. This test involves both classical fear conditioning and instrumental responses and is known to be hippocampus-dependent (Ho et al., 2019; Mihaylova et al., 2019). The AAT is not frequently used to assess the cognitive functions of experimental SAH animals; however, we compared the ATT results with those of the Morris Water Maze (MWM) test obtained in our previous experiment and found that the two tests had similar ability in assessing cognitive function in SAH rats. Two measures from the AAT, i.e., motor function score and the ratio of ITIR number to trial number (ITIR%), were used to determine whether physical deficits were present that could confound the results of the cognitive test. The results showed that the motor function of rats was impaired at 24 h after SAH but had recovered by day 4 during the AAT, consistent with most previous studies reporting no evidence of motor deficits in cognitive tests 2–4 days after SAH (Jeon et al., 2009; Smithason et al., 2012). Our study showed that compared with sham rats, SAH rats performed worse in the AAT and that treatment with NaHS improved the learning and memory ability of rats with SAH. H_2_S has been regarded as the third endogenous signaling gasotransmitter, with nitric oxide and carbon monoxide being the other two. H_2_S production was significantly downregulated by SAH in the rat brain, and treatment with NaHS (an H_2_S donor) restored the brain H_2_S content (Cui et al., 2016). Researchers have found that H_2_S attenuated cognitive impairment in an AD model (Xuan et al., 2012), cerebral ischemic disease (Li et al., 2011), an LPS-induced cognitive impairment model (Gong et al., 2010), a traumatic brain injury model (Karimi et al., 2017), and a SAH model (Li et al., 2017). However, the mechanisms underlying the effects of H_2_S on cognitive function have not been identified; possible effects of H_2_S on neuroinflammation (Liu et al., 2015), antioxidant effects, glutamate and NMDA receptors (Chen et al., 2017), phosphatidylinositol 3-kinase (PI3-K)/Akt signaling pathway have been proposed (He et al., 2019).

The mechanism underlying cognitive impairment after SAH remains unclear as well. In our experiment, histology of the hippocampus revealed no apparent neuron necrosis or neutrophil infiltration in any of the groups, consistent with a previous report (Tariq et al., 2010). This finding suggests that cognitive impairment is unlikely to be due to cell death or neuroinflammation induced by neutrophil infiltration in the hippocampus, at least in this model. However, we found that SAH indeed enhanced neuroinflammation in the hippocampus, as evidenced by the enhanced expression of TNF-α and the activation of microglia, and induced cognitive impairment in rats. Both hippocampal neuroinflammation and cognitive impairment were alleviated by NaHS, which indicated a relationship between neuroinflammation and cognitive deficits. H_2_S may reduce cognitive impairment by ameliorating neuroinflammation in microglia. Neuroinflammation has been proven to be closely associated with cognitive impairment in neurodegenerative diseases, and a few studies have shown that neuroinflammation could worsen the cognitive impairment caused by SAH (Provencio et al., 2016). Learning and memory impairment can occur without obvious neuronal death (Gong et al., 2010) or neutrophil infiltration into the hippocampus after SAH (Provencio et al., 2016). As microglia are the major immune cells in the CNS (Hanisch and Kettenmann, 2007), their activation may be responsible for neuroinflammation-driven cognitive impairment.

We also explored the probable pathway underlying the neuroinflammation and found that the expression of cytokines associated with the TLR4/NF-κB pathway was upregulated after SAH but downregulated by H_2_S. Furthermore, double immunofluorescence staining revealed that the cell types expressing TNF-α mostly colocalized with TLR4, indicating that H_2_S probably attenuated neuroinflammation mediated by the TLR4/NF-κB pathway. Studies have found that the TLR4/NF-κB pathway might be an important therapeutic target for post-SAH neuroinflammation (Okada and Suzuki, 2017). TLR4 is one of the common pattern-recognition receptors (PRRs) expressed in various cell types in CNS, mainly in microglia, and can be activated by both PAMPs and DAMPs (Kawasaki and Kawai, 2014). In SAH, the breakdown products of red blood cells could be the main DAMPs (Lucke-Wold et al., 2016). DAMPs in SAH activate TLR4 on microglia and induce microglia and other glia to become the main sources of cytokines in the CNS. Activation of TLR4 leads to downstream activation of NF-κB, which is known to upregulate the expression of proinflammatory cytokines such as TNF-α, chemokines, adhesion molecules, and acute-phase proteins. These cytokines, in turn, activate microglia and other glia, playing a key role in the induction and maintenance of neuroinflammation (Sochocka et al., 2017).

However, it remains unclear how neuroinflammation affects cognitive impairment. In AD brains, elevated levels of proinflammatory cytokines, including TNF-α, inhibited the phagocytosis of Aβ; this inhibition may be responsible for the cognitive deficits in AD (Xuan et al., 2012). Related information in SAH is lacking, although the mechanism in SAH seems to differ from that in AD. Some evidence supports the role of inflammation in the pathogenesis of cerebral vasospasm (CVS; Provencio et al., 2011), a common and potentially devastating complication of SAH that leads to cerebral ischemia and may contribute to cognitive impairment. Also, loss of late long-term potentiation (L-LTP) and dysfunction of the NMDA receptor, which are associated with inflammation, have been found in SAH (Provencio et al., 2016). Our study suggests that activation of microglia and elevated levels of inflammatory cytokines may be key mechanisms. H_2_S reduced cognitive impairment after SAH by inhibiting the inflammation induced by activated microglia. Microglia is the most important resident immune cell within the CNS and typically has a ramified appearance under normal physiological conditions. Ramified microglia are generally described as “resting” but are not functionally inert. Recent research identified roles of the fine processes of ramified microglia in synaptic plasticity, cognition, and disease, which, along with other glial cells such as astrocytes, are intimately involved in the complexities of neural networks and memory formation. Microglia can be activated by cell injury and pathogen infection. Activated microglia are morphologically characterized by de ramification, changing from highly ramified to amoeboid cells with thickened processes and enlarged cell bodies (Provencio et al., 2011; Wolf et al., 2017). These changes are consistent with our finding that the amoeboid microglia, which were most abundant in the SAH group, expressed TNF-α, while the ramified microglia, which were mainly observed in the SAH + NaHS group, did not (Morris et al., 2013). Glial dysfunction following brain injury due to morphological and physiological changes can alter mechanisms of synaptic plasticity and is associated with an increased risk for learning and memory deficits (Sajja et al., 2016). Moreover, recent studies suggest that cytokines such as TNF-α are present in the healthy brain and play important roles in controlling synaptic transmission and plasticity. Increased TNF-α levels transform the physiological actions of cytokines into adverse ones, which may contribute to cognitive deficits (Santello and Volterra, 2012).

Several limitations of the present study should be noted. First, no significant effects of H_2_S on the neurological deficits of rats after SAH were found, differing from the report by Cui et al. (2016). One of the probable reasons for this difference is the different scoring systems employed in the two studies: that of Cui mainly evaluated appetite, activity, and neurological defects, being a more specialized neurological scoring system than necessary for SAH. Second, although the present study found that H_2_S attenuated post-SAH cognitive impairment, probably by ameliorating neuroinflammation, and identified microglial activation and enhanced TNF-α expression as the potential mechanisms by which inflammation affects cognitive function, the relationships between neuroinflammation and other factors, such as glutamate and NMDA receptors, were not explored. These relationships need to be elucidated in future studies.

## Conclusion

Our results demonstrated that: (1) neuroinflammation in microglia contributed to cognitive impairment after SAH; and (2) H_2_S reduced cognitive impairment in rats after SAH, probably by ameliorating the neuroinflammation mediated by the TLR4/NF-κB pathway in microglia.

## Data Availability Statement

The raw data supporting the conclusions of this article will be made available by the authors, without undue reservation.

## Ethics Statement

The protocol of this study was reviewed and approved by the Ethics Committee of Peking University First Hospital and following the guidelines of the National Institutes of Health on the care and use of animals.

## Author Contributions

HD, LL, and SS contributed to the conception and design of the study. HD and SS conducted most of the experiments, and HD wrote the first draft of the manuscript. YM made the SAH model in the experiment. XY, ZL, CY, YW, and JZ did immunohistochemical staining, Western blot, data collection, and analysis during the experiment. LL and JZ revised the manuscript. All authors read and approved the submitted manuscript.

## Conflict of Interest

The authors declare that the research was conducted in the absence of any commercial or financial relationships that could be construed as a potential conflict of interest.
